# Antimicrobial Properties of *Daucus nebrodensis* Strobl.: A Multifunctional Essential Oil Against Bacterial Pathogens

**DOI:** 10.3390/plants14142227

**Published:** 2025-07-18

**Authors:** Giusy Castagliuolo, Antonella Porrello, Maddalena Cerasola, Giuseppe Bazan, Dario Antonini, Mario Varcamonti, Maurizio Bruno, Anna Zanfardino, Natale Badalamenti

**Affiliations:** 1 Department of Biology, University of Naples Federico II, 80126 Naples, Italyvarcamon@unina.it (M.V.); 2 Department of Biological, Chemical and Pharmaceutical Sciences and Technologies (STEBICEF), Università degli Studi di Palermo, Viale delle Scienze, ed. 17, 90128 Palermo, Italymaurizio.bruno@unipa.it (M.B.);; 3 National Biodiversity Future Center (NBFC), 90133 Palermo, Italy

**Keywords:** Nebrodi mountains, *Apiaceae*, sabinene, antibiotic resistance, antibiofilm activity

## Abstract

*Daucus* is a large genus of the Apiaceae family, comprising around forty-five accepted species, that has a worldwide distribution. Species of this genus have been reported to have several traditional medicinal uses, and some of them are also largely used as food and spices. *Daucus nebrodensis* Strobl. is an endemic species of Sicily growing in the montane environments of the Madonie and the Nebrodi Mountains. In this work, the essential oil of *D. nebrodensis* (*Dn*EO), collected wild near Messina (Italy), was chemically and biologically investigated. The hydrodistilled essential oil (yield 0.15%), obtained from fresh aerial parts, was evaluated by GC-MS, and It was particularly rich in monoterpene hydrocarbons, with sabinene (33.6%), α-pinene (17.2%), *γ*-terpinene (9.8%), and α-terpinene (7.6%) as the main metabolites. *Dn*EO, and its main constituents, have been tested to evaluate their biological properties. Given the current problem of antibiotic resistance, it is of great interest to identify alternative molecules that could counteract the its progression. Therefore, *Dn*EO was tested against Gram-negative species, such as *E. coli* DH5α and *P. aeruginosa* PAOI, and Gram-positive species, such as *S. aureus* ATCC6538P, *B. subtilis* AZ54, and *M. smegmatis* MC^2^155, showing notable antibacterial activity. The MIC for *Bacillus subtilis*, the most sensitive strain, was 18 mg/mL, while the MIC for *Pseudomonas aeruginosa*, the least sensitive strain, was 30 mg/mL. Moreover, interesting antibiofilm activity was observed against *Mycobacterium smegmatis* with a 55% inhibition. Its ability to form biofilms contributes to its persistence and resistance in clinical settings. These findings highlight the potential of *D. nebrodensis* EO as a source of bioactive compounds with promising antimicrobial and antibiofilm properties.

## 1. Introduction

*The Daucus* genus, belonging to the *Apiaceae* family, includes forty-five accepted species [1] distributed worldwide. In fact, although the Mediterranean area has been considered the center of diversity for this genus, many species are geographically distributed in other continents such as Africa, Australia, and America [2,3].

Species belonging to the *Daucus* genus, as well as other species of the *Apiaceae* family are well known for their use due to their biological properties [4], but it is also notable that several taxa of this genus are economically significant as food sources. In particular, *Daucus carota* L. subsp. *sativus* Hoffm. is common for its use as a vegetable, and it represents the most cultivated species of the *Daucus* genus. This species, among the most studied of the *Daucus* genus, is also known for its ethnobotanical applications. For example, the infusion of leaves or flowers has been used to alleviate disorders of the reproductive system, but also against urinary problems and diabetes [5]. All parts of the plant show useful application; in Turkey, the seeds of *D. carota* are employed for the treatment of gastrointestinal and respiratory disorders, while in Chinese culture the fruits are utilized for the management of ascariasis, enterobiasis, and tapeworm disease [6]. In this regard, traditional use of *D. carota* has attracted attention in regard to investigating the proposed pharmacological properties, not only of the latter but also in other species belonging to the genus [7,8,9,10].

Other taxa belonging to the genus *Daucus* have also been investigated due to the presence of bioactive compounds of interest for their potential pharmacological applications. Among the reported activities for plants of this genus, essential oils (EOs) and extracts have shown several properties such as antioxidant [11], antimicrobial [12], fungicidal [13], carminative, diuretic, hepatoprotective [14], anti-steroidogenic [15], and anti-inflammatory [14,16]. Regarding antibacterial properties, for example, the EOs of *D. syrticus*, characterized by higher monoterpene hydrocarbons, show remarkable antimicrobial activity against four microbes with MIC values between 0.5 and 1.0 [17]. On the other hand, not only the EOs of *Daucu*s but also the extracts were tested to evaluate some biological effects, such as the hydrosol extracts of *D. muricatus*, which showed potential use as sources of natural antioxidants due to the presence of oxygenated monoterpenes and phenylpropanoids [18].

In Sicily, several species of *Daucus* occur, including not only several subspecies of *D. carota* but also *D. aureus* Desf., *D. muricatus* (L.) L., and *D. nebrodenis* Strobl., the object of this work [19].

**Figure 1:**
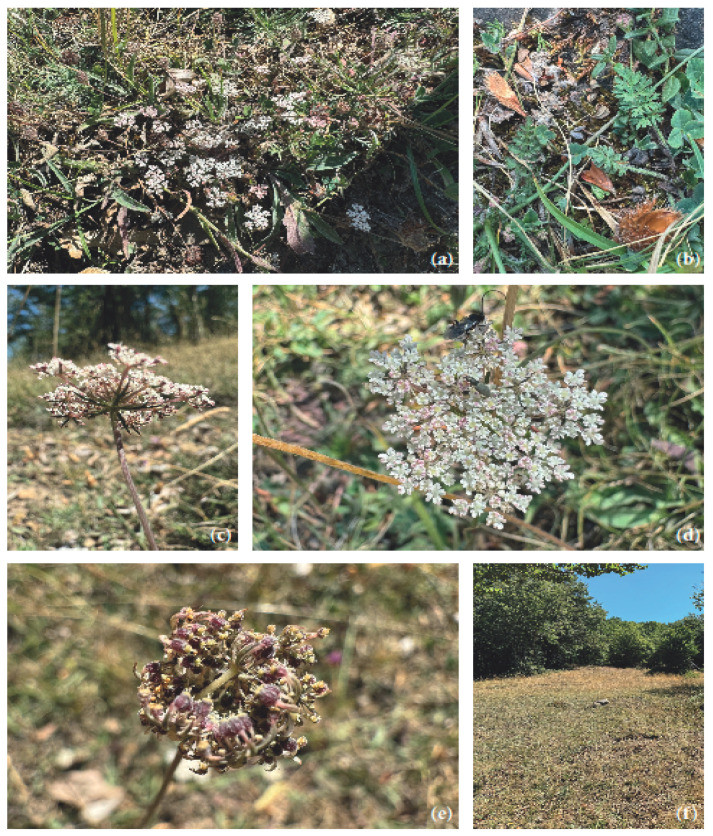
(**a**) *Daucus nebrodensis*, the whole plant in its natural habitat; (**b**) close-up of the leaves; (**c**) bracts and bracteoles during the flowering stage; (**d**) radiant umbels, white in color, with some slightly pinkish; (**e**) fruiting stage with ovoid achenes; (**f**) species habitat in mesophilous grasslands.

*Daucus nebrodensis* Strobl. is a biennial hemicryptophyte reaching a maximum height of 30 cm, with numerous slender (Figure 1a), almost glabrous branches emerging from the base of a slightly hispid stem.

The leaves are lanceolate in outline (Figure 1b), with short pinnate leaflets; the lower ones are cuneate-ovate, while the upper ones are linear-lanceolate, with petioles bearing patent hairs. The bracts are initially almost as long as the umbel but, at the fruiting stage, reach barely half the umbel’s length. They are rarely divided into three bifurcate lobes and are usually entire and linear-lanceolate (Figure 1c). The bracteoles of the involucel are linear and simple. The umbels are small and short but relatively well-radiated (Figure 1d), bearing flowers with styles three times longer than the stylopodium and divergent. The petals are white, rarely pinkish, and rounded. The fruits are ovoid achenes (Figure 1d), barely 3 mm in length, with numerous white setae measuring only 0.5 mm [20].

An endemic species of Sicily, *D. nebrodensis* has been recorded in the montane environments of the Madonie [21], the Nebrodi Mountains, and on the slopes of Mount Etna above Nicolosi [22]. Within its distribution range, the species grows in mesophilous grasslands and pastures (Figure 1f) at elevations above 1000 m, within the supramediterranean bioclimatic zone [23], with an ombrotype ranging from lower subhumid to lower humid. The taxon is not rare within its distribution range and is classified as Near Threatened (NT) [24].

*D. nebrodensis* is listed in the most recent checklist of the Italian vascular flora [25]. However, the authors note that this species may not be distinctly separated from *Daucus carota* subsp. *hispanicus* (Gouan) Thell. Furthermore, World Flora Online reports *D. nebrodensis* as a synonym of *D. carota* subsp. *hispanicus* [26]. However, the latter taxon is a coastal morphotype [27], reported in the Flora of Italy [28] as *D. gingidium* L. subsp. *fontanesii* (Thell.), commonly known as the “cliff carrot,” and recorded in Sicily in coastal habitats [22].

To date no research has been reported for *D. nebrodensis*. Considering this gap, the present work aims to characterize the profile of the EO derived from a Sicilian accession of *D. nebrodensis* and to evaluate its antimicrobial, antibiofilm, and antioxidant activities.

Interest in plant-derived EOs has grown considerably in recent years, particularly for their potential role in addressing antimicrobial resistance. These natural products are rich sources of bioactive compounds, many of which exhibit bactericidal or bacteriostatic properties [29,30].

Moreover, several volatile constituents have been identified as inhibitors of bacterial biofilm formation and persistence, offering promising alternative or complementary approaches to conventional antibiotics [31,32].

The *Daucus* genus, which belongs to the *Apiaceae* family, is well-known for its numerous biological properties, among which an interesting antimicrobial activity stands out. This activity is mainly attributed to the EO compounds found in various parts of the plants. Specifically, several studies have highlighted that the EOs of certain *Daucus* species possess a broad antimicrobial spectrum, effective against both Gram-positive and Gram-negative bacteria. Another example of *Daucus* biological potential is the study conducted by Dib et al. [33]: in this case the solvent extracts from the leaves, stems, and roots of *Daucus crinitus* Desf. were studied revealing significant antimicrobial activity against various pathogenic bacteria, such as *Bacillus cereus, Staphylococcus aureus, Escherichia coli,* and *Candida albicans*.

The most well-known member of this genus, *D. carota*, has been extensively studied for its antimicrobial properties, primarily attributed to the bioactive compounds in its EOs. The chemical composition and antimicrobial activity of the EOs of *D. carota* vary significantly depending on the subspecies and collection location, with the identification of volatile compounds such as monoterpenes (e.g., α-pinene, sabinene, and γ-terpinene) and sesquiterpenes, which act through multiple mechanisms, including disruption of cell membranes, loss of cytoplasmic contents, and impairment of bacterial cell vital functions [34,35].

A study conducted on *D. carota* highlighted the EO′s strong antibacterial activity, particularly against Gram-positive strains like *S. aureus*, suggesting that the simpler cell wall structure of these bacteria makes them more susceptible to the action of the EOs [36]. Moreover, a good correlation was observed between the chemical composition of the oils and their antimicrobial activity, emphasizing the importance of the synergy among the different components in expressing the overall effectiveness of the EO [37].

Some studies have also explored the synergistic potential of *Daucus* EOs as adjuvants in antimicrobial therapies, showing that mixtures of EOs from different *Daucus* species can enhance the inhibitory effect compared to isolated single components. This phenomenon is attributed to complex interactions between secondary metabolites, which can alter cell permeability and facilitate intracellular absorption of active agents, thereby improving therapeutic efficacy [38].

Other studies have examined the composition of *D. carota* EOs from various geographical locations. In Tunisia, an analysis of *D. carota* seeds identified 36 compounds, including β-bisabolene (39.33%), sabinene (8.53%), geranyl acetate (7.12%), and elemicin (6.26%), which exhibited antibacterial activity against *S. aureus, E. coli, Salmonella typhimurium*, and *C. albicans* [39]. Another study identified (E)-methylisoeugenol and elemicin as the active compounds in *D. carota* EOs, showing good antimicrobial activity against strains of *Campylobacter jejuni*, *Campylobacter coli,* and *Campylobacter lari*, including multi-resistant strains [40].

In Tunisia, EOs isolated from D. carota using supercritical CO_2_ showed variable compositions: those from Sejnane contained eudesm-7 (11)-en-4-ol (8.2–8.5%), carotol (3.5–5.2%), sabinene (12.0–14.5%), and α-selinene (7.4–8.6%), while those from Tunis were rich in elemicin (31.5–35.3%) and carotol (48.0–55.7%). However, antimicrobial activity against *E. coli, S. aureus,* and *Candida* strains was limited, with MIC values exceeding 2.5% (*v*/*v*) [41].

In parallel, the antioxidant potential of EOs has been widely reported. Their efficacy is largely attributed to the presence of phenolic and monoterpenoid compounds, capable of scavenging free radicals and mitigating oxidative stress-features with significant implications in both nutraceutical and pharmaceutical applications [42,43].

By providing the first data on the chemical composition and biological potential of *D. nebrodensis*, this study seeks to enhance the scientific understanding and valorization of this underexplored Sicilian endemic species.

## 2. Results and Discussion

### 2.1. Chemical Composition of DnEO

Hydro-distillation of *D. nebrodensis* flowering aerial parts gave a yellow EO (*Dn*EO, yielded 0.15%). The total composition was identified for 95.6%, and twenty-five compounds were found. The components are listed in Table 1 according to their retention indices on a DB-5 MS column and are classified based on their chemical structures into four different classes.

The *Dn*EO profile was essentially composed of monoterpene hydrocarbons (84.8%), mainly represented by sabinene (33.6%), *α*-pinene (17.2%), *γ*-terpinene (9.8%), and *α*-terpinene (7.6%). 4-Terpineol (5.7%) was the most abundant compound belonging to oxygenated monoterpenes (7.9%). Sesquiterpene hydrocarbons and oxygenated sesquiterpenes were present in very limited amounts, representing, 0.7% and 2.2%, respectively.

Several reports have been published on the chemical composition of the EOs from the aerial part of *Daucus* species [34,45,46,47,48,49,50,51]. A comparison of *Dn*EO investigated in this work with those reported in the literature shows some very interesting considerations.

Specifically, sabinene, the principal metabolite of *Dn*EO, was also found to be a major compound in EOs from the aerial part of *D. setifolius* [45], *D. aureus* Desf. [46], *D. muricatus* [12,18,47], and in EOs obtained from other vegetative parts of *D. carota* ssp., *D. carota* ssp. *carota* [48,49], *D. carota* ssp. *maritimus* [50], and *D. carota* ssp. *halophilus* [34]; but unlike these, limonene, which is present in good amounts in many of the latter species, is totally absent in *Dn*EO, the subject of this study. However, the *Dn*EO profile shows a relative composition characterized by a very high content of hydrocarbon monoterpenes, which is consistent with that reported in the literature for most *Daucus* EOs.

The second most abundant constituent of *Dn*EO was *α*-pinene, which represents 17.2% of the total composition. This compound, within its isomer *β*-pinene (also present in *Dn*EO at 2.3%), is very common in the composition of *Daucus* EOs. Indeed, they are occurring in almost all *Daucus* species currently described in the literature.

Regarding the remaining two significant compounds, *α*-terpinene and *γ*-terpinene, their occurrence is comparatively limited. Indeed, *γ*-terpinene was identified in several EOs; in particular, this compound represents one of the principal constituents of the *D. syrticus* [17] aerial part EO, despite also being found in the root of *D. carota* ssp. *maximus* [43] and flowers of *D. carota* ssp. *maritimus* [41], highlighting the ubiquitarian nature of this monoterpene in the different parts of plants of the *Daucus* genus. As for *α*-terpinene, it was present only in *D. syrticus* [17] aerial parts EO, representing 4.4% of the composition.

Nevertheless, a small part of the EOs from *Daucus* taxa has a particular composition characterized by the presence of myristicin, which was not identified in the *Dn*EO sample. *D. carota* ssp. *hispanicus* aerial part EO shows a level of myristicin ranging between 66.9 and 83.8%, while a lower amount (16.6%) has been identified in the roots [13]. Myristicin was found in good amounts in *D. sahariensis* [52,53,54], *D. carota* ssp. *gummifer* [55], *D. carota* ssp. *maritimus* [50,55], *D. carota* ssp. *sativus* [56], *D. glaber* [57], *D. guttatus* ssp. *zahariadii* [58], and *D. littoralis* ssp*. hyrcanicus* [59].

In summary, although the composition of *Dn*EO appears to be consistent with that found for other EOs of plants belonging to the same genus, the coexistence of the four majority compounds present in the above-mentioned EO has never been found previously in any other *Daucus* species, showing a peculiarity of *Dn*EO. Further studies on other stations of *Daucus nebrodensis* could be useful to confirm what is reported in this work.

### 2.2. Antimicrobial Properties of DnEO

This study examined the antimicrobial activity of the EO isolated from *Daucus nebrodensis* (*Dn*EO), an endemic and not-studied species collected in the Madonie mountains in Sicily. This species is characterized by a unique EO composition, rich in α-pinene, α-terpinene, sabinene, and *γ*-terpinene.

**Figure 2:**
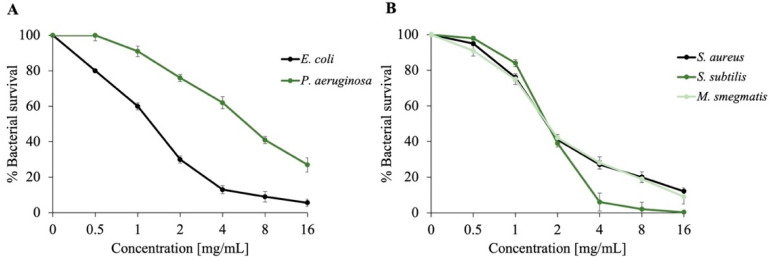
Evaluation of the antimicrobial activity of *Dn*EO at varying concentrations, assessed through colony-forming unit (CFU) counting assays against Gram-negative (**A**) and Gram-positive strains (**B**). Bacterial survival is expressed on the y-axis as a percentage, calculated by comparing the CFU counts of treated samples to those of untreated controls. All experiments were performed in three independent biological replicates; standard deviations remained consistently below 10%.

The analysis of its antibacterial activity was performed against both Gram-positive and Gram-negative bacterial strains, using viable cell count assays. Figure 2 presents the dose–response curves obtained by varying the *Dn*EO concentration and evaluating bacterial survival relative to controls. Gram-negative strains (*E. coli* and *Pseudomonas aeruginosa*), involved in intestinal and respiratory infections, and Gram-positive strains (*S. aureus, Bacillus subtilis*, and *M. smegmatis*), associated with skin and systemic infections, were selected.

As shown in Figure 2, there is a clear inverse relationship between the concentration of *Dn*EO and the bacterial survival rate. Overall, *Dn*EO demonstrated antimicrobial activity against both Gram-positive and Gram-negative strains. Notably, *E. coli* exhibited a marked dose–response trend, suggesting a relatively high sensitivity under the tested conditions, comparable to that observed for Gram-positive strains. In contrast, *P. aeruginosa* showed a more resistant profile. These observations align with previous studies where various essential oils rich in α-pinene and sabinene exhibited greater effectiveness against Gram-positive bacteria [49,60].

To quantify the antimicrobial activity, three independent experiments were conducted to determine the MIC using the broth microdilution method. As shown in Table 2, Gram-positive strains exhibited the highest sensitivity, with the lowest MIC values ranging from 18 to 25 mg/mL. In contrast, Gram-negative strains showed higher MIC values, ranging from 25 to 30 mg/mL, indicating comparatively lower susceptibility. These findings confirm a greater effectiveness of the EO against Gram-positive bacteria, in agreement with previous studies on essential oils rich in α-pinene and sabinene. Notably, these results are promising when compared to MIC values reported for other Daucus species. To the best of our knowledge, no quantitative MIC data have been previously reported for *D. nebrodensis* essential oil, underscoring the novelty and significance of this study.

**Figure 3:**
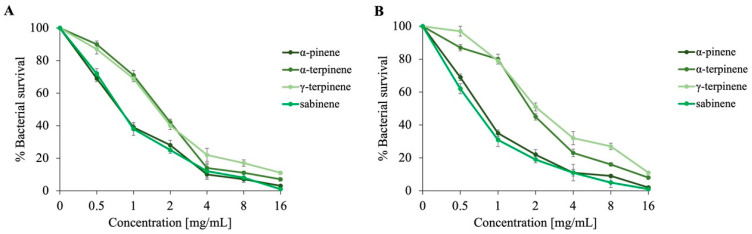
Evaluation of the antimicrobial activity of individual components of *Dn*EO (*α*-pinene, *α*-terpinene, sabinene, and *γ*-terpinene) at different concentrations, evaluated through colony-forming unit (CFU) assays against *E. coli* (**A**) and *S. aureus* (**B**). Bacterial survival is shown on the *y*-axis as a percentage, calculated by comparing the CFU counts of treated samples to those of untreated controls. All experiments were performed in 3 independent biological replicates, with standard deviations consistently below 10%.

Since the chemical profile of *Dn*EO was found to include specific volatile compounds such as *α*-pinene, *α*-terpinene, sabinene, and *γ*-terpinene, the contribution of each component was explored by testing them individually at the same relative concentrations found in *Dn*EO. Figure 3 illustrates the dose–response curves obtained by testing individual volatile compounds against *E. coli* (Figure 3A) and *S. aureus* (Figure 3B). Among the compounds analyzed, *α*-pinene and sabinene demonstrated the strongest antimicrobial effects, as evidenced by the steeper decline in bacterial survival observed at concentrations as low as 1 mg/mL, compared to the other tested components.

To provide a quantitative assessment of their activity, the minimum inhibitory concentration (MIC) of the individual *Dn*EO compounds was subsequently determined. The MIC values confirmed the pronounced efficacy of *α*-pinene and sabinene, with values of 23 mg/mL and 20 mg/mL against *S. aureus* and 25 mg/mL and 24 mg/mL against *E. coli* (Table 3). These results agree with those reported by Bassolé and Juliani [37], who highlighted the remarkable antimicrobial potency of both compounds against a wide range of microbial strains. Overall, these results are consistent with those obtained from other *Daucus* species and further support the hypothesis that the antimicrobial activity of their EOs arises from synergistic or antagonistic interactions between multiple volatile constituents.

### 2.3. Mechanistic Evaluation of DnEO and Its Major Compounds Against Bacterial Target

**Figure 4:**
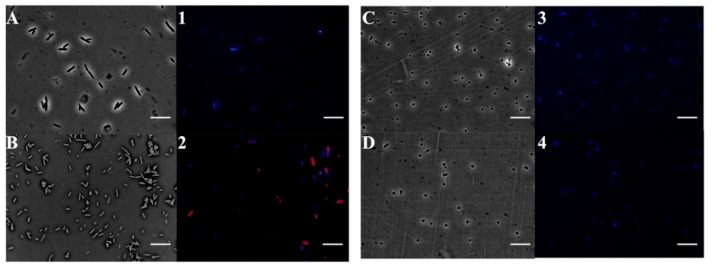
Fluorescence microscopy-based analysis of the antimicrobial mode of action of *Dn*EO. The panels display *E. coli* cells (**A**,**B**-**1**,**2**) and *S. aureus* cells (**C**,**D**-**3**,**4**). (**A**–**D**) show cells imaged under bright-field microscopy, while (**1**–**4**) show the corresponding fluorescence images. Untreated cells are shown in (**A**-**1**,**C**-**3**); *Dn*EO treated cells are shown in (**B**-**2**,**D**-**4**). Scale bars: 5 µm.

To investigate the mechanism of action of *Dn*EO and its main components, fluorescence microscopy experiments were carried out. The aim was to assess their effect on bacterial membrane integrity using *E. coli* and *S. aureus* cells stained with DAPI and PI. DAPI is a DNA-binding fluorescent dye that emits blue light, while PI emits red fluorescence and only enters cells with compromised membranes. Therefore, PI serves as an indicator of membrane damage. As shown in Figure 4 (Panels A-1 and C-3), untreated control cells appeared intact and fluoresced blue due to DAPI staining. Notably, after 4 h of treatment with sub-MICs of *Dn*EO, *E. coli* cells (Panel 2) exhibited red fluorescence, suggesting membrane disruption. In contrast, *S. aureus* cells treated under the same conditions did not show red fluorescence, implying that in Gram-positive bacteria, the primary target may differ from the cell membrane. These results suggest that *Dn*EO may exert its antimicrobial effect on Gram-negative bacteria through membrane damage, whereas a different mode of action may be involved in Gram-positive strains.

**Figure 5:**
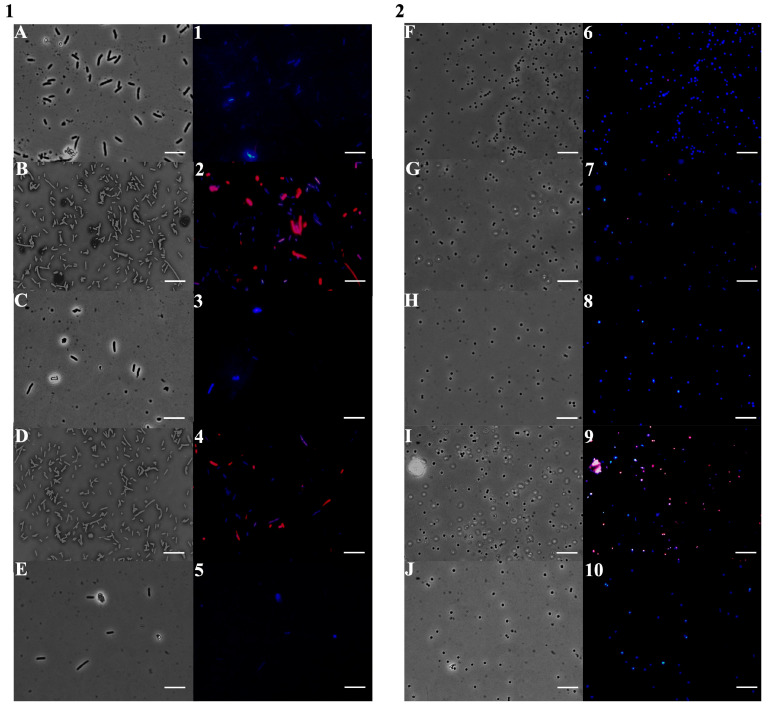
Fluorescence microscopy analysis of the antimicrobial mode of action of selected components of *Dn*EO on *E. coli* (**1**) and *S. aureus* (**2**). Bright-field images (**A**–**J**) are paired with their corresponding fluorescence images (**1**–**10**). Untreat-ed control cells are shown in (**A-1**,**F-6**); cells treated with α-pinene in (**B-2**,**G-7**), α-terpinene in (**C-3**,**H-8**), sabinene in (**D-4**,**I-9**), and γ-terpinene in (**E-5**,**J-10**). Scale bars: 5 µm.

To further explore the cellular target, we stained both *E. coli* and *S. aureus* cells with DAPI/PI after treatment with individual *Dn*EO components, applied at concentrations equivalent to those found by GC-MS. As shown in Figure 5, untreated cells (Panel 1, A-1; Panel 2, F-6) displayed only blue fluorescence, indicating intact membranes. The same was observed in cells treated with *α*-terpinene and *γ*-terpinene (Panel 1: C-3, E-5; Panel 2: H-8, J-10), suggesting these compounds do not disrupt the bacterial membrane. Interestingly, cells treated with α-pinene and sabinene (Panel 1: B-2, D-4; Panel 2: G-7, I-9) showed strong red fluorescence, indicating significant membrane damage. This points to a possible role of *α*-pinene and sabinene in compromising membrane integrity, especially in the case of *E. coli* and sabinene against *S. aureus.*

The analysis of bacterial membrane integrity following treatment with *Dn*EO and its individual components provided significant evidence of the complex interplay between the phytochemical constituents and their biological activity. While sub-MICs of the whole *Dn*EO resulted in only mild membrane permeabilization, limited to *E. coli*, treatment with selected individual components, particularly *α*-pinene and sabinene, resulted in marked membrane disruption in both *E. coli* and *S. aureus*, as evidenced by strong red fluorescence in the propidium iodide (PI) channel.

This important result raises crucial questions about the dynamics of synergy and antagonism within EO blends. Despite the use of each compound in its relative abundance in the total oil, their isolated application revealed a greater potential for disruption of bacterial membranes. A plausible explanation is the presence of antagonistic interactions in the whole EO, where less active or inactive components could interfere with or neutralize the effects of *α*-pinene and sabinene [61,62]. Such interference may occur through competitive binding to membrane components, reduced diffusion or solubility, or even physical entrapment within micellar structures, ultimately limiting the accessibility of active compounds to the bacterial surface [63]. Furthermore, some EO constituents may exert membrane-stabilizing effects, counteracting the disruptive action of terpenes. This phenomenon is well documented in the context of complex phytochemical mixtures, where secondary metabolites may interact in multiple ways, giving rise to emergent properties that cannot be predicted by individual activity alone [64]. It is also conceivable that whole *Dn*EO, as a complex chemical matrix, may undergo altered partitioning or adsorption dynamics when applied to bacterial cultures, influencing the local concentration and bioavailability of active compounds on the membrane. The increased susceptibility of *S. aureus* and *E. coli* to membrane damage induced by isolated *α*-pinene and sabinene further supports previous findings highlighting the potent antimicrobial action of these monoterpenes. However, the total (Gram-positive) and partial (Gram-negative) suppression of this effect in the context of the total oil highlights the importance of analyzing both individual components and whole extracts to better understand their bioactive properties and limitations. These findings underscore the need to carefully evaluate interactive effects among EO’s constituents, as the net antimicrobial effect may not simply reflect the sum of the parent compounds′ activities. Rather, the final effect results from a complex balance of synergistic, additive, and antagonistic interactions, which may enhance or diminish the biological efficacy of the mixture.

### 2.4. Investigation of the Antibiofilm Activity of DnEO

**Figure 6:**
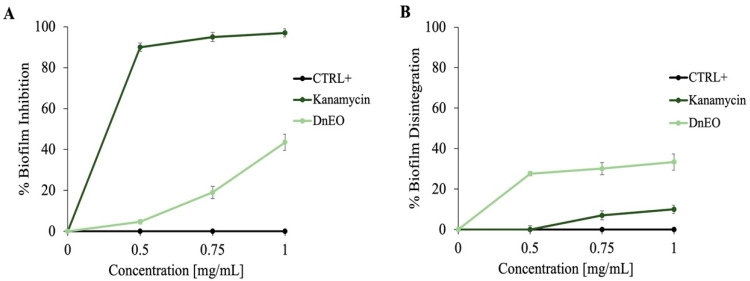
Colorimetric evaluation of *M. smegmatis* biofilm inhibition and disintegration at varying concentrations of *Dn*EO. (**A**) illustrates the percentage of biofilm formation inhibition, while (**B**) displays the percentage of performed biofilm disintegration. Untreated cells served as a positive control, and kanamycin-treated cells as a negative control. All assays were conducted in triplicate, with standard deviations consistently below 10%.

To our knowledge, this is the first study to explore the potential antibiofilm properties of *Dn*EO. Given the well-documented activity of several EOs against biofilm formation at low concentrations [65], we hypothesized that *Dn*EO might exhibit similar effects. To test this hypothesis, experiments using *M. smegmatis*, a nonpathogenic model organism frequently employed in mycobacterial research due to its genetic and physiological similarities to *Mycobacterium tuberculosis,* were conducted. Biofilm formation by *M. smegmatis* is of particular interest, as it plays a crucial role in bacterial persistence and antibiotic resistance, mirroring the problems observed in more pathogenic mycobacterial species. Because the minimum inhibitory concentration (MIC) of *Dn*EO against *M. smegmatis* was previously determined to be 25 mg/mL, we tested concentrations much lower than the MIC (0 to 1 mg/mL), at which no bacterial mortality occurs, to assess both inhibition and biofilm destruction. As shown in Figure 6, panel A, treatment with *Dn*EO at 1 mg/mL resulted in approximately 55% inhibition of biofilm formation. This is a noteworthy result, as it suggests that *Dn*EO could interfere with the early stages of biofilm development, a key process in bacterial colonization and persistence of infection.

Furthermore, the oil′s ability to disrupt preformed biofilms, a more challenging task due to the structural and chemical resilience of mature biofilm matrices, was evaluated. As illustrated in Figure 6, panel B, *Dn*EO induced a biofilm disaggregation of around 40%, indicating a promising dual action: both preventative and curative in nature. This dual functionality is particularly valuable in clinical and environmental settings, where established biofilms are often resistant to conventional antimicrobial treatments. These findings highlight the potential of *Dn*EO as a natural anti-biofilm agent, with possible applications in the development of novel treatments targeting chronic infections where biofilms play a central role.

**Figure 7:**
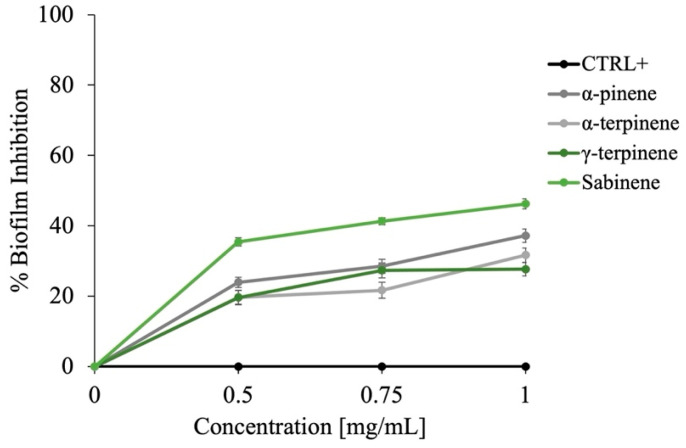
Colorimetric evaluation of *M. smegmatis* biofilm inhibition at varying concentrations of *α*-pinene, *α*-terpinene, *γ*-terpinene, and sabinene. Untreated cells served as a positive control. l assays were conducted in triplicate, with standard deviations consistently below 10%.

In addition, the role of individual components of *Dn*EO in inhibiting biofilm formation, was investigated. As shown in Figure 7, *α*-pinene and sabinene demonstrated the greatest capacity to inhibit biofilm formation, with inhibition percentages ranging between 37% and 46%. This result is particularly interesting because it confirms, as previously reported in the fluorescence experiments, the high potential of these two components, reflecting the fact that the overall biofilm inhibition by the total *Dn*EO oil was approximately 55%. It is likely that the action of these two components largely contributes to the anti-biofilm effect observed with the complete sample.

### 2.5. Study of the Antioxidant Properties of DnEO and Its Components

Recent investigations into the antioxidant activity of EOs have revealed that their free radical scavenging abilities—specifically against DPPH and ABTS radicals—are highly dependent on both the concentration of the EO and its chemical composition, particularly the profile of its major constituents [66]. In the case of the *Dn*EO, its dominant compounds —*α*-pinene, *α*-terpinene, sabinene, and *γ*-terpinene—belong to the class of monoterpenes, a group of naturally occurring hydrocarbons, widely recognized for their significant role in antioxidant mechanisms. These compounds are not oxygenated terpenes but hydrocarbon-based terpenes, and their antioxidant capacity has been linked to their ability to donate hydrogen atoms or electrons to neutralize free radicals [67].

**Figure 8:**
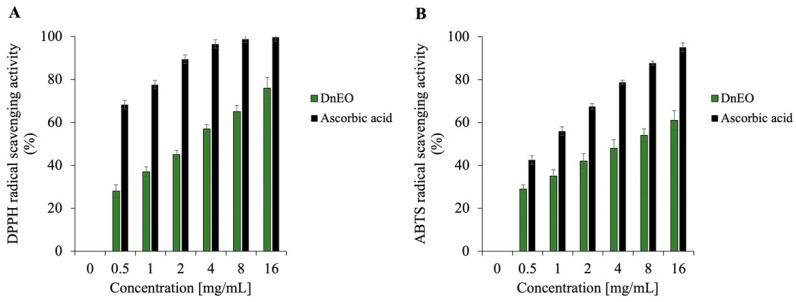
Assessment of the antioxidant activity of *Dn*EO. (**A**) presents the DPPH radical scavenging capacity after 30 min of incubation, expressed as the percentage of DPPH reduction. (**B**) shows the ABTS radical scavenging activity, measured after 10 min and expressed as the percentage of ABTS reduction. The positive control for both assays is ascorbic acid, used at the same concentrations. Data represent the mean values from three independent experiments.

As shown in Figure 8, both DPPH (panel A) and ABTS (panel B) assays demonstrate a concentration-dependent increase in radical scavenging activity of *Dn*EO across the tested range (0–16 mg/mL). The DPPH assay, a commonly employed method for assessing the ability of a substance to donate electrons and stabilize free radicals, indicates that *Dn*EO possesses robust antioxidant potential, achieving approximately 80% radical scavenging at the highest tested concentration. Similarly, the ABTS assay—which evaluates a compound’s capacity to neutralize highly reactive cationic radicals—confirmed a substantial antioxidant effect of about 50%, suggesting the *Dn*EO contains bioactive molecules capable of attenuating oxidative stress by interacting with reactive oxygen species (ROS).

**Figure 9:**
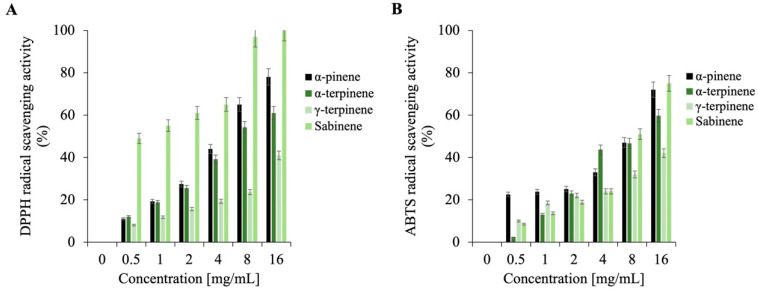
Assessment of the antioxidant activity of major individual components of *Dn*EO. (**A**) displays the DPPH radical scavenging activity of *α*-pinene, *α*-terpinene, sabinene, and *γ*-terpinene after 30 min of incubation, expressed as the percentage of DPPH reduction relative to the control. (**B**) illustrates the ABTS scavenging activity of the same compounds, measured after 10 min and reported as the percentage of ABTS reduction compared to the control. Results are presented as the means of three independent biological replicates.

Given that antioxidant capacity in EOs is generally attributed to their chemical composition, the role of *α*-pinene, *α*-terpinene, sabinene, and *γ*-terpinene was investigated. As illustrated in Figure 9 (DPPH: panel A; ABTS: panel B), sabinene and *α*-pinene displayed particularly potent activity at 16 mg/mL, with DPPH scavenging rates of 100% and 98%, respectively, and approximately 70% scavenging in the ABTS assay. Ascorbic acid was also used as a positive control for this assay, but the data are not reported as they are the same as those shown in Figure 7. These findings clearly indicate that these two terpenes are major contributors to the antioxidant potential observed in the whole *Dn*EO sample.

Interestingly, *Dn*EO did not achieve the same level of activity as its individual components, despite being tested at concentrations reflecting their natural proportions in the oil sample. This discrepancy suggests possible antagonistic interactions among the components of the EO matrix. Such antagonism might occur when the presence of one compound interferes with the radical-scavenging efficiency of another, potentially through competition for active sites, steric hindrance, or modulation of redox potential in the complex mixture. These findings highlight the importance of understanding not only the individual activity of EO constituents but also their interactive behavior within the complete phytochemical profile, which can significantly influence the overall biological effect.

### 2.6. Assessment of Cell Viability by MTT Assay

**Figure 10:**
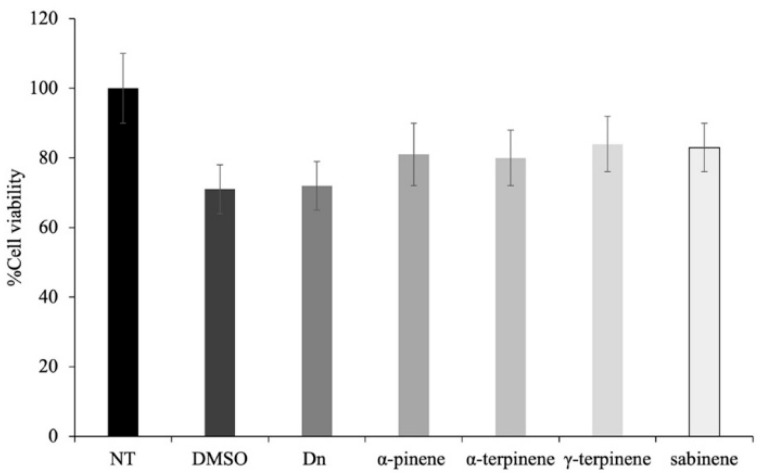
Cytotoxic effect of *Dn*EO and of the *α*-pinene, *α*-terpinene, *γ*-terpinene, and sabinene components on eukaryotic cells. HaCat cells were treated for 4 h. Cellular cytotoxicity was determined using the MTT assay. The untreated cells (NT) were assumed to be 100%. The percentage of cell survival in each condition was calculated by comparing with the untreated cells. The assays were performed in three independent experiments. The error bars indicate the standard error of the mean (SEM).

To verify whether the *Dn*EO and the main compounds (*α*-pinene, *α*-terpinene, *γ*-terpinene, and sabinene) found within it could be toxic to eukaryotic cells, human keratinocytes were used to perform an assay using the MTT assay. Interestingly, both *Dn*EO and the other compounds upon 4 h of treatment at the 16 mg/mL concentration are not cytotoxic (Figure 10).

These findings suggest that *Dn*EO and its major constituents are not cytotoxic at the tested concentration and exposure time, indicating a favorable safety profile for potential topical or therapeutic applications. This result is particularly relevant in the context of antimicrobial agent development, where selective toxicity toward microbial cells over host cells is a key requirement.

## 3. Materials and Methods

### 3.1. Plant Materials

The fresh and flowering aerial parts from *Daucus nebrodensis* were collected on Mt. Soro, (Messina, Sicily, Italy (37°55′48,432″ N; 14°40′29,058″ E), in spring 2025. One of the samples, identified by Prof. Giuseppe Bazan, has been stored in the Herbarium Mediterraneum Panormitanum (PAL), (Voucher N.109876), of the Botanical Garden of the University of Palermo, Italy.

### 3.2. Isolation of EO

Fresh flowers, leaves, and stems (780 g) from ten plants were subjected to hydrodistillation for 3 h, according to the standard procedure described in the European Pharmacopoeia [68]. Samples yielded 0.15% of EO.

### 3.3. GC-MS Analysis

Analysis of EO was carried out according to the procedure reported by Lauricella et al. [69]. GC-MS analyses were carried out using a Shimadzu QP 2010 Plus system equipped with an AOC-20i auto-injector (Shimadzu, Kyoto, Japan), and a non-polar capillary column (DB-5 MS, 30 m × 0.25 mm i.d., 0.25 μm film thickness), along with a data processor. The oven temperature program was as follows: initial hold at 40 °C for 5 min, followed by a ramp of 2 °C per minute up to 260 °C, and then maintained isothermally for 20 min. Helium served as the carrier gas at a flow rate of 1 mL/min. The injector and detector temperatures were set to 250 °C and 290 °C, respectively. A 1 μL aliquot of the essential oil solution (3% EO in hexane, *v*/*v*) was injected in split mode (1.0); the mass scan range was 40–600 *m*/*z*. Instrumental parameters included an ionization voltage of 70 eV, an electron multiplier voltage of 2000 V, a transfer line temperature of 295 °C, and a solvent delay of 3 min. Linear retention indices (LRIs) were calculated using a mixture of pure *n*-alkanes (C_8_–C_40_), and all the peaks’ compounds were identified by comparison with MS and by comparison of their relative retention indices with WILEY275, NIST 17, ADAMS, and FFNSC2 libraries.

### 3.4. Microbial Strain Selection

The antimicrobial activity of *Dn*EO and its principal individual constituents was evaluated against a panel of bacterial strains, including Gram-negative species, such as *E. coli* DH5α and *P. aeruginosa* PAOI, and Gram-positive species, such as *S. aureus* ATCC6538P, *B. subtilis* AZ54, and *M. smegmatis* MC^2^155.

### 3.5. Assessment of Antibacterial Properties

To assess the antimicrobial activity of *Dn*EO, a cell viability assay was performed using both Gram-positive and Gram-negative bacterial strains. Microbial cultures were exposed to *Dn*EO at concentrations ranging from 0.5 to 16 mg/mL and incubated at 37 °C for 4 h. Following incubation, samples were serially diluted and plated on agar Petri dishes to determine viable colony-forming units (CFUs). Untreated bacterial cultures served as positive controls, while cells treated with 50% DMSO, used as a solvent for the essential oil, were included as negative controls. Colony counts were performed the following day, and bacterial survival rates were calculated relative to the positive controls [70]. The assay was also conducted on the main individual constituents of the EO—*α*-pinene (17.2%), α-terpinene (7.6%), sabinene (33.6%), and *γ*-terpinene (9.8%)—tested at the same concentration range as the whole *Dn*EO. These compounds, however, were evaluated against two representative model organisms: *E. coli* (Gram-negative) and *S. aureus* (Gram-positive). All experiments were conducted in triplicate, and results are presented as the mean values of three independent replicates.

### 3.6. Evaluation of Minimum Inhibitory Concentration (MIC)

The minimum inhibitory concentrations (MICs) of *Dn*EO against the selected bacterial strains were determined using the microdilution method recommended by the Clinical and Laboratory Standards Institute (CLSI) [71]. Bacterial suspensions at a final concentration of 5 × 10^5^ CFU/mL were added to 95 µL of Mueller-Hinton broth (CAM-HB; Difco), supplemented with varying concentrations of the EO and of the individual components, ranging from 1 to 35 mg/mL.

Following overnight incubation at 37 °C, MIC values were defined as the lowest concentration of the oil at which no visible bacterial growth was observed, as determined by measuring optical density at 600 nm. The same procedure was applied to *S. aureus* and *E. coli* to evaluate the activity of the individual EO constituents. All experiments were performed in triplicate, and results are expressed as the mean of three independent assays.

### 3.7. Fluorescence-Based Viability Assay Using DAPI and PI

To assess bacterial membrane integrity via fluorescence microscopy, two stains were employed: DAPI (4′, 6-diamidino-2-phenylindole dihydrochloride; Sigma-Aldrich, Milan, Italy) and propidium iodide (PI; Sigma-Aldrich, Milan, Italy). Briefly, 100 µL aliquots of bacterial cultures (*E. coli* and *S. aureus* model strains) were incubated in the dark at 37 °C for 4 h under shaking conditions, either in the presence or absence of *Dn*EO or its major components *α*-pinene, *α*-terpinene, sabinene, and *γ*-terpinene, at sub-MICs.

Following incubation, 10 µL of each culture was mixed with a staining solution containing DAPI (1 µg/mL) and PI (20 µg/mL). Samples were then examined under an Olympus BX51 fluorescence microscope (Olympus, Tokyo, Japan) equipped with a DAPI filter set (excitation/emission: 358/461 nm). The standard exposure time for DAPI/PI dual staining was set to 1000 ms. Images were captured using an Olympus DP70 digital camera, following the protocol described by Di Napoli et al. [72].

### 3.8. Biofilm Disruption and Inhibition Assays

The antibiofilm activity of *Dn*EO was evaluated against *M. smegmatis* biofilms using a colorimetric assay. Untreated microbial cells served as positive controls, while cultures treated with kanamycin (2 µg/mL) were used as negative controls. A 24-well plate was prepared and incubated at 37 °C for 72 h in the presence of *Dn*EO and the individual components, at concentrations ranging from 0 to 1 mg/mL, to assess its ability to inhibit biofilm formation. A second, identically prepared plate was incubated without *Dn*EO to allow biofilm development; *Dn*EO was then added after 72 h to assess its capacity to disrupt pre-formed biofilms, followed by an additional 24 h incubation. At the end of each incubation period, crystal violet staining was performed to quantify biofilm biomass. Optical density (OD) measurements were taken at 570 nm using a Multiskan microplate reader (Thermo Electron Corporation, Waltham, MA, USA), according to established protocols [73]. The percentage of biofilm inhibition or disruption was calculated by comparing the OD values of treated samples with those of untreated controls. To account for potential differences in planktonic cell growth, OD values at 570 nm were normalized by calculating the ratio between the absorbance at 570 nm and the corresponding OD at 600 nm, thus allowing for a more accurate comparison of biofilm formation across different conditions.

### 3.9. DPPH and ABTS Scavenging Capacity Assay

The antioxidant activity of *Dn*EO and its main components, *α*-pinene, *α*-terpinene, sabinene, and *γ*-terpinene, was evaluated using DPPH and ABTS radical scavenging assays, following the protocol described by Napolitano et al. [74]. A range of concentrations (0–16 mg/mL) for each sample was prepared in a final reaction volume of 1 mL.

For the DPPH assay, freshly prepared DPPH solution (0.1 mM in 100% methanol) was used, adjusted to yield an initial absorbance of ≤1.0. In the ABTS assay, ABTS radical cations were generated by mixing 7 mM ABTS with 2.45 mM potassium persulfate, and the solution was then diluted with phosphate-buffered saline (PBS) to an absorbance of approximately 0.72. Reaction mixtures were incubated at room temperature (30 min for DPPH and 10 min for ABTS) before absorbance was measured at 517 nm and 734 nm, respectively. Ascorbic acid, a positive control, has been used in the same quantities of essential oil.

Radical scavenging capacity was expressed as a percentage of inhibition, calculated using the equation:

Scavenging activity (%) = [1 – (AS/AC)] × 100

where AS represents the absorbance of the sample (*Dn*EO or individual component, normalized to the blank) after reaction with the radical solution, and AC denotes the absorbance of the radical solution alone.

### 3.10. Cell Viability Assay

The cytotoxic activity of *Dn*EO and its components (*α*-pinene, *α*-terpinene, *γ*-terpinene, and sabinene) was evaluated using the MTT assay. Cells were seeded in 96-well microplates and incubated for 24 h at 37 °C. Then, the plates were incubated for 4 h with DMSO (negative control), *Dn*EO or *α*-terpinene, *γ*-terpinene and sabinene at the concentration of 18 mg/mL. To assess the cell survival, 10 µL of MTT in 90 µL of DMEM solution was added to each well, and the plates were incubated at 37 °C for 4 h. Then, the media were removed, and 100 µL of DMSO was added to each well and incubated for 10 min. Absorbance was measured at 570 nm using a Synergy H4 Hybrid Microplate reader (Agilent, Santa Clara, CA, USA). Each concentration was assayed in four wells and repeated three times. Cell viability was calculated based on the untreated cells that were assumed as 100%.

## 4. Conclusions

This study provides the first in-depth characterization of the antimicrobial, antibiofilm, and antioxidant activities of the essential oil from *Daucus nebrodensis*, an endemic and little-studied species from Sicily. Essential oil was obtained from fresh aerial parts, and its chemical profile, characterized by GC-MS analysis, shows the presence of twenty-five compounds, with *α*-pinene, sabinene, *α*-terpinene, and *γ*-terpinene as principal components. The results demonstrate that *Dn*EO possesses significant antimicrobial activity, particularly against Gram-positive bacteria, with MIC values ranging between 18 and 25 mg/mL. Specifically, *B. subtilis* was the most sensitive strain, with an MIC value of 18 mg/mL. A similar trend was observed with the individual components: the Gram-positive model strain *S. aureus* showed MIC values of 23 mg/mL for α-pinene and 20 mg/mL for sabinene. In fact, the analyses of the individual components revealed that *α*-pinene and sabinene are the main contributors to the antimicrobial effect and bacterial membrane permeabilization. However, the overall efficacy of the oil appears to be modulated by complex interactions among its constituents, suggesting the presence of both synergistic and antagonistic effects. Additionally, *Dn*EO exhibited promising antibiofilm activity, both preventive and curative, against *M. smegmatis*, and showed strong antioxidant potential mainly attributed to sabinene and *α*-pinene. Finally, treatment with *Dn*EO and its major components did not show cytotoxic effects in human keratinocytes, indicating a favorable preliminary safety profile. These findings suggest that *Daucus nebrodensis* may represent a valuable natural source for the development of novel antimicrobial and antioxidant agents.

## Figures and Tables

**Table 1:** Composition (%) of *Daucus nebrodensis* EO (*Dn*EO) collected in Sicily.

No.	Compounds ^a^	LRI ^b^	LRI ^c^	Area (%)
1	*α*-Thujene	925	924	2.6
2	*α*-Pinene *	930	936	17.2
3	Camphene	942	947	1.0
4	Sabinene *	972	969	33.6
5	*β*-Pinene	990	990	2.3
6	*α*-Phellandrene	1000	1006	1.7
7	*α*-Terpinene *	1013	1021	7.6
8	Dolcymene	1020	1023	1.0
9	Sylvestrene	1025	1029	5.0
10	*β*-*trans*-Ocimene	1037	1040	0.3
11	*β-cis-*Ocimene	1047	1050	0.2
12	*γ*-Terpinene *	1057	1056	9.8
13	Terpinolene	1085	1087	2.5
14	Linalool	1097	1101	0.3
15	4-Thujanol	1115	1114	0.4
16	Allo-Ocimene	1127	1134	1.0
17	4-Terpineol	1173	1180	5.7
18	*α*-Terpineol	1185	1189	0.2
19	Isobornyl acetate	1279	1290	0.2
20	Eugenol methyl ether	1399	1401	0.1
21	*β-*Eudesmene	1479	1476	0.5
22	Shyobunone	1487	1491	1.4
23	Isohomogenol	1492	1495	0.4
24	*β*-Bisabolene	1505	1503	0.2
25	*α*-Bisabolol	1680	1680	0.4
	Monoterpene Hydrocarbons			84.8
	Oxygenated Monoterpenes			7.9
	Sesquiterpene Hydrocarbons			0.7
	Oxygenated Sesquiterpenes			2.2
	Total			95.6

^a^ Components listed in order of elution on a DB-5 MS apolar column; ^b^ Experimental LRIs on a DB-5 MS apolar column. ^c^ LRIs based on the literature [44]. * Major compounds > 7.0%.

**Table 2:** Determination of the minimum inhibitory concentration (MIC, expressed in mg/mL) of *Dn*EO against Gram-negative and Gram-positive bacterial strains. MIC values were derived from at least three independent biological replicates.

Strains	MIC [mg/mL]
*E. coli*	25
*P. aeruginosa*	30
*S. aureus*	20
*B. subtilis*	18
*M. smegmatis*	25

**Table 3:** Determination of the minimum inhibitory concentration (MIC, expressed in mg/mL) of individual components of *α*-pinene, *α*-terpinene, sabinene, and *γ*-terpinene against *E. coli* and *S. aureus*. MIC values were derived from at least three independent biological replicates.

MIC [mg/mL]				
	*α*-pinene	*α*-terpinene	*γ*-terpinene	sabinene
*E. coli*	25	30	>35	24
*S. aureus*	23	30	35	20

## Data Availability

All data and materials are available upon request from the corresponding author.
